# Information bottleneck-based Hebbian learning rule naturally ties working memory and synaptic updates

**DOI:** 10.3389/fncom.2024.1240348

**Published:** 2024-05-16

**Authors:** Kyle Daruwalla, Mikko Lipasti

**Affiliations:** ^1^Cold Spring Harbor Laboratory, Long Island, NY, United States; ^2^Electrical and Computer Engineering Department, University of Wisconsin-Madison, Madison, WI, United States

**Keywords:** neuromorphic computing, Neural Network, learning rule, information bottleneck, back-propagation

## Abstract

Deep neural feedforward networks are effective models for a wide array of problems, but training and deploying such networks presents a significant energy cost. Spiking neural networks (SNNs), which are modeled after biologically realistic neurons, offer a potential solution when deployed correctly on neuromorphic computing hardware. Still, many applications train SNNs *offline*, and running network training directly on neuromorphic hardware is an ongoing research problem. The primary hurdle is that back-propagation, which makes training such artificial deep networks possible, is biologically implausible. Neuroscientists are uncertain about how the brain would propagate a precise error signal backward through a network of neurons. Recent progress addresses part of this question, e.g., the weight transport problem, but a complete solution remains intangible. In contrast, novel learning rules based on the information bottleneck (IB) train each layer of a network independently, circumventing the need to propagate errors across layers. Instead, propagation is implicit due the layers' feedforward connectivity. These rules take the form of a three-factor Hebbian update a global error signal modulates local synaptic updates within each layer. Unfortunately, the global signal for a given layer requires processing multiple samples concurrently, and the brain only sees a single sample at a time. We propose a new three-factor update rule where the global signal correctly captures information across samples via an auxiliary memory network. The auxiliary network can be trained *a priori* independently of the dataset being used with the primary network. We demonstrate comparable performance to baselines on image classification tasks. Interestingly, unlike back-propagation-like schemes where there is no link between learning and memory, our rule presents a direct connection between working memory and synaptic updates. To the best of our knowledge, this is the first rule to make this link explicit. We explore these implications in initial experiments examining the effect of memory capacity on learning performance. Moving forward, this work suggests an alternate view of learning where each layer balances memory-informed compression against task performance. This view naturally encompasses several key aspects of neural computation, including memory, efficiency, and locality.

## 1 Introduction

The success of deep learning demonstrates the usefulness of large feedforward neural networks for solving a variety of tasks, but the energy cost associated with such networks presents an ongoing problem (Strubell et al., 2019). Neuromorphic computing platforms and spiking neural networks (SNNs), which model the power efficient properties of neural networks, offer a possible solution (Christensen et al., 2022). While recent advances allow SNNs to be trained *offline* (Neftci et al., 2019), these approaches only benefit from energy-efficient inference even though training continues to be the dominant energy bottleneck for deep learning. Though there are many strategies for training SNNs, it is widely believed that the most effective technique will be a biologically plausible learning rule (Zenke et al., 2021). While reproducing biology is not a strict requirement, the engineering constraints of neuromorphic hardware naturally align with biological constraints. Namely, we identify three defining properties of biologically plausible learning rules that directly impact energy efficiency: *locality, asynchrony, and real-time processing*. These three properties reduce the communication overhead and coordination required by a neuromorphic chip which are large sources of power consumption (Christensen et al., 2022).

Unfortunately, training spiking neural networks directly on hardware is challenging, since the driving factor behind deep learning's success—back-propagation—is not considered to be biologically plausible (Lillicrap et al., 2020). Specifically, it is unclear how neurons might propagate a precise error signal within a forward/backward pass framework like back-propagation. A large body of work has been devoted to establishing plausible alternatives or approximations for this error propagation scheme (Balduzzi et al., 2015; Scellier and Bengio, 2017; Akrout et al., 2019; Lillicrap et al., 2020). While these approaches do address some of the issues with back-propagation, implausible elements, like separate inference and learning phases, still persist in many cases.

**Figure 1:**
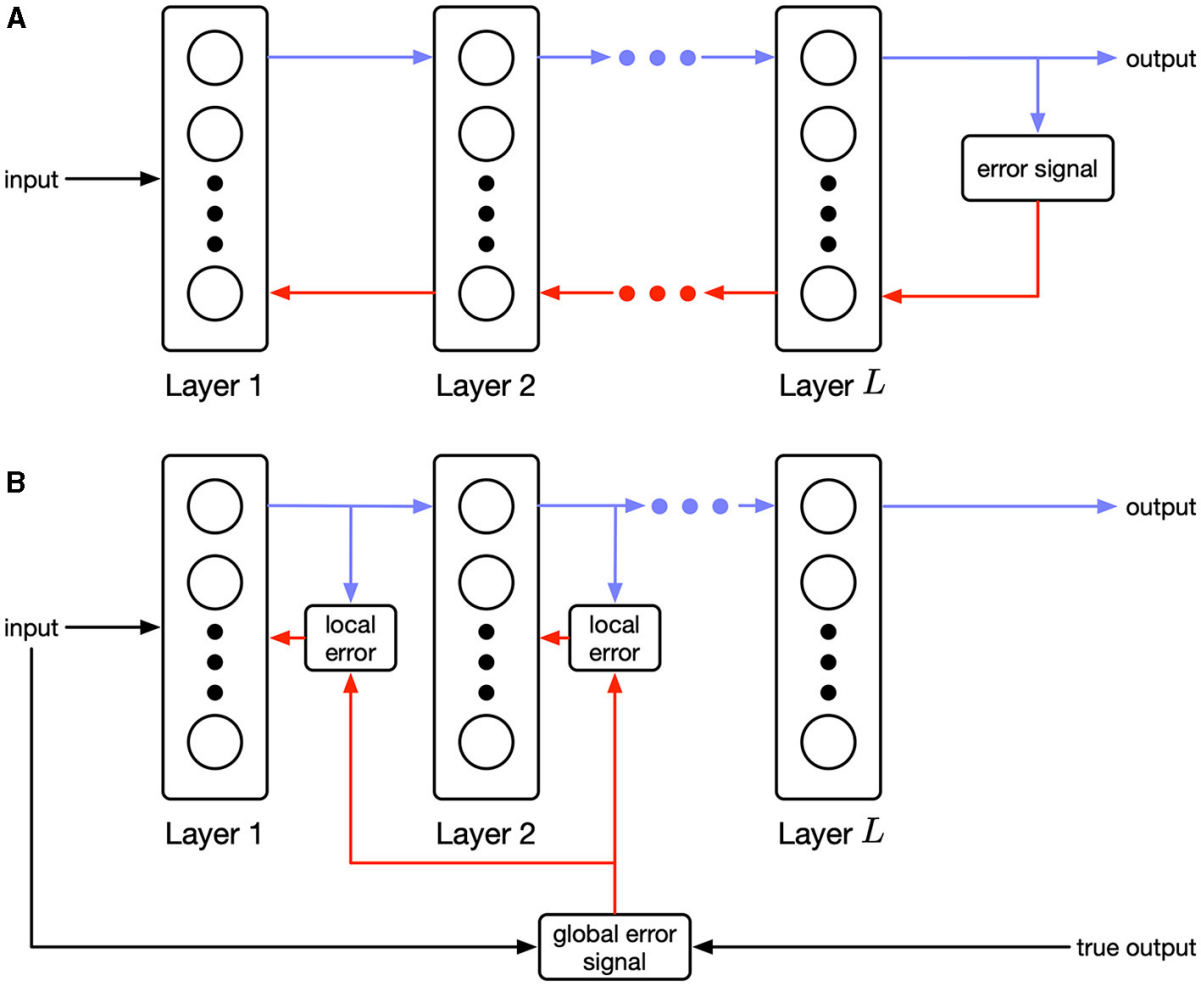
**(A)** Sequential (explicit) error propagation requires precise information transfer *backwards* between layers. **(B)** Parallel (implicit) error propagation uses only local information in combination with a global modulating signal. Biological rules of this form are known as three-factor learning rules (Frémaux and Gerstner, 2016).

Our work joins a body of recent literature that addresses biological plausibility by suggesting fundamentally different approaches to training networks from back-propagation (Payeur et al., 2021; Meulemans et al., 2022; Aceituno et al., 2023). These approaches modulate local Hebbian updates using top-down signals based on alternative objectives such as optimizing a control policy. Our work is similar in that we propose a dramatically different training objective. In contrast, we rely on recent advances in deep learning that train feedforward networks by balancing an information bottleneck objective (Ma et al., 2019). Unlike back-propagation, where an error signal computed at the end of the network is propagated to the front (see Figure 1A), this method, called the Hilbert-Schmidt Independence Criterion (HSIC) bottleneck, applies the information bottleneck to each layer in the network independently. Layer-wise optimization is biologically plausible as shown in Figure 1B. Compared to related work, where the performance of the final layer affects training of prior layers through top-down signals, our objective is fully localized at each layer.

Our contributions include:

1. We show that optimizing the HSIC bottleneck via gradient descent emits a three-factor learning rule (Frémaux and Gerstner, 2016) composed of a local Hebbian component and a global layer-wise modulating signal.
2. The HSIC bottleneck depends on a batch of samples, and this is reflected in our update rule. Unfortunately, the brain only sees a single sample at a time. We show that the local component only requires the current sample, and that the global component can be accurately computed by an auxiliary network. The auxiliary networks acts as a working memory with post-processing, and the effective “batch size” corresponds to its capacity.
3. We demonstrate the empirical performance of our update rule by comparing it against baselines on synthetic datasets as well as MNIST (LeCun et al., 1998) and CIFAR-10 (Krizhevsky, 2009).
4. To the best of our knowledge, our rule is the first to make a direct connection between working memory and synaptic updates. We explore this connection in some initial experiments on memory size and learning performance.

### 1.1 Preliminaries and related work

Several works have presented approximations to back-propagation. Variants of feedback alignment (Lillicrap et al., 2014; Liao et al., 2016; Akrout et al., 2019) address the weight transport problem. Target propagation (Ahmad et al., 2020; Frenkel et al., 2021) and equilibrium propagation (Scellier and Bengio, 2017) propose alternative mechanisms for propagating error. Yet, all these methods require separate inference (forward) and learning (backward) phases. More recently, deep feedback control methods (Meulemans et al., 2022; Aceituno et al., 2023) use top-down signaling from a controller to optimize forward and backward weights concurrently. Unlike prior methods which address biological plausibility piecemeal, these techniques are plausible by design. We follow this approach to creating plausible learning rules, but we differ by focusing on layer-wise objectives instead of top-down control. Table 1 shows a comprehensive comparison between learning rule definitions. Only direct feedback control (DFC) and our work satisfy all objectives, but they represent two different solutions to the problem of biologically plausible learning rules. DFC is framed as a control problem with continuous dynamics, and the resulting weight update requires multi-compartment neurons. The authors note that this makes their work better suited for analog neuromorphic hardware. In contrast, our rule can be mapped to both digital or analog hardware, since time is denoted by sequences of samples and not physical time. Additionally, we do not put constraints on the neurons required to implement the rule.

**Table 1:** A comparison of various learning algorithms categorized by four properties.

**Learning algorithm**	**Weight transport-free?**	**Local?**	**Asynchronous?**	**Real-time?**
Back-propagation (BP)	✗	✗	✗	✗
Feedback alignment (FA)	✓	✗	✗	✗
Direct FA (DFA)	✓	✓	✗	✗
Single sparse DFA (SDFA)	✓	✓	✓	✗
Equilibrium propagation (EP)	✓	✓	✗	✗
Target propagation (TP)	✓	✓	✗	✗
Direct random TP (DRTP)	✓	✓	✓	✗
Plausible HSIC (pHSIC)	✓	✓	✓	✗
Direct feedback control (DFC)	✓	✓	✓	✓
Our work	✓	✓	✓	✓

*Weight transport-free* rules do not require separate forward and backward networks with aligned weight parameters. *Local* rules utilize only locally available information. *Asynchronous* rules do not require a full forward pass before updating the weights of each layer. *Real-time* rules operate on samples arriving sequentially in time (not batches).

Layer-wise objectives (Belilovsky et al., 2019; Nøkland and Eidnes, 2019), like the one used in this work, offer an alternative that avoids the weight transport problem entirely. Moreover, our objective emits a biologically plausible three-factor learning rule which can be applied concurrently with inference. Pogodin and Latham (2020) draw similar intuition in their work on the plausible HSIC (pHSIC) learning rule. But in order to make experiments with the pHSIC computationally feasible, the authors used an approximation where the network receives a batch of 256 samples at once. In contrast, their proposed biologically plausible rule only receives information from two samples—the current one and previous one—which reduces the accuracy of the HSIC estimate. This motivates our work, in which we derive an alternate rule where only the global component depends on past samples, while the local component only requires the current pre- and post-synaptic activity. Furthermore, we show that this global component can be computed using an auxiliary network. This allows us to achieve performance much closer to back-propagation without compromising the biological plausibility of the rule.

#### 1.1.1 Other uses of information theoretic objectives for spiking neural networks

Information bottleneck and other information theoretic quantities have been used in the context of training SNNs before. Yang and Chen (2023a,b) utilize an information bottleneck objective in the final layer of an SNN to train networks that are robust to noisy input distributions. Yang and Chen (2023a) improves on the standard information bottleneck by considering higher order terms. Similarly, Yang et al. (2022) trains networks with an additional minimum entropy criterion to promote robust learning in SNNs. Still, all works rely on back-propagation through time (BPTT) and surrogate gradient descent to train their SNNs.

#### 1.1.2 Hardware substrates for implementing biological neural networks

While our work does not directly deal with hardware implementations of biological networks, our contributions are motivated by the possible power efficiency benefits of biologically plausible learning rules. As such, we will briefly discuss various platforms for physical realization of neuromorphic computing.

The landscape of neuromorphic hardware is vast and varied. At one extreme, platforms like Intel's Loihi (Davies et al., 2018, 2021) use conventional CMOS technologies to create a digital array of biological neurons. While such systems are useful for exploring SNN applications, it is widely accepted that the primary power efficiency of neuromorphic hardware will come from novel device technologies. The most common devices are memristors (Yan et al., 2023) and resistive memory (Bianchi et al., 2023). Less common substrates based on metal-organic transistors (Wang et al., 2023) and thermal-guiding structures (Loke et al., 2016) exist as well. These devices are designed to mimic various functions of a biological synapse especially its plastic conductance. While the specifics differ, all devices have electrical properties that allow the conductance to be adjustable. Successful demonstrations of neuromorphic device arrays show their ability to simulate SNNs at a much lower power consumption than conventional computing. Yet, all rely on purely local update rules which fail to scale to very deep networks. Circumventing this limitation requires non-local circuitry, and the goal of any biologically plausible rule, including ours, is to limit the power overhead of these components.

#### 1.1.3 Notation

We will briefly introduce the notation used in the paper.

- Vectors are indicated in bold and lower-case (e.g., **x**).
- Matrices are indicated in bold and upper-case (e.g., **W**).
- Superscripts refer to different layers of a feedforward network (e.g., **z**^ℓ^ is the ℓ-th layer).
- Subscripts refer to individual samples (e.g., **x**_*i*_ is the *i*-th sample).
- Brackets refer to elements within a matrix or vector (e.g., [**x**]_*i*_ is the *i*-th element of **x**).

## 2 Methods and materials

In this section, we describe our learning rule and its derivation in detail. Section 2.1 introduces the information bottleneck for deep networks. Then in Section 2.2, we derive a gradient descent rule for this objective, as well as introduce reasonable approximations such that the final rule is a three-factor Hebbian update. Lastly, in Section 2.2.1 we describe how the modulating factor in our rule can be computed using an auxiliary network.

### 2.1 The information bottleneck

Given an input random variable, *X*, an output label random variable, *Y*, and hidden representation, *T*, the information bottleneck (IB) principle is described by Equation (1)


(1) $$\begin{array}{l} \underset{{\mathbb{P}}_{T|X}}{\min}I\left(X;T\right)-\gamma I\left(Y;T\right) \end{array}$$

where *I*(*A*; *B*) is the mutual information between two random variables. Intuitively, this expression adjusts *T* to achieve a balance between information compression and output preservation.

Since computing the mutual information of two random variables requires knowledge of their distributions, Ma et al. (2019) propose using the Hilbert-Schmidt Independence Criterion (HSIC) as a proxy for mutual information. Given a finite number of samples, *N*, a statistical estimate, shown in Equation (2), for the HSIC (Gretton et al., 2005) is


(2) $$\begin{array}{l} HSIC(X,Y)={\left(N-1\right)}^{-2}\text{tr}(K_{X}HK_{Y}H)\\ \;\leq \frac{1}{{\left(N-1\right)}^{2}}\sum_{p=1}^{N}\bar{k}(x_{p},x_{p})\bar{k}(y_{p},y_{p}) \end{array}$$

(3) $$\begin{array}{l} \left[K_{X}H]t_{pq}\;=\bar{k}(x_{p},x_{q}\right)\\ \;=k(x_{p},x_{q})-\frac{1}{N}\sum_{n=1}^{N}k(x_{p},x_{n}) \end{array}$$

(4) $$\begin{array}{l} {\left[{\text{K}}_{\text{X}}\right]}_{pq}=k\left({\text{x}}_{p},{\text{x}}_{q}\right)=\exp\left(-\frac{||{\text{x}}_{p}-{\text{x}}_{q}|{|}^{2}}{\sigma^{2}}\right) \end{array}$$

where Equations (3) and (4) define the centered and uncentered kernel matrices, respectively.

Using these definitions Ma et al. (2019) define the HSIC objective—a loss function for training feedforward neural networks by balancing the IB at each layer. Consider a feedforward neural network with *L* layers where the output of layer ℓ is


$${\text{z}}^{\ell }=f\left(\theta^{\ell },{\text{z}}^{\ell -1}\right)$$

where *f* describes the forward computation (including nonlinear activation) of a single layer given parameters, **θ**^ℓ^, and inputs, **z**^ℓ−1^. For example, a fully-connected layer of artificial neurons with a ReLU activation is described by **θ**^ℓ^ = {**W**^ℓ^, **b**^ℓ^} and *f*(**θ**^ℓ^, **z**^ℓ−1^) = relu(**W**^ℓ^**z**^ℓ−1^+**b**^ℓ^). In this work we will define *f* for both artificial neuron layers and rate-encoded leaky-integrate neuron layers.

We train the network to minimize


(5) $$\begin{array}{l} {ℒ}_{\text{HSIC}}(X,Y,Z^{\ell })=\text{HSIC}(X,Z^{\ell })-\text{HSIC}(Y,Z^{\ell })\\ \;\forall \ell \in \{1,\ldots ,L\} \end{array}$$

where $Z={\left\{Z^{\ell }\right\}}_{\ell =1}^{L}$ are the output distributions at each hidden layer. Note that there is a separate objective for each layer. As a result, there is no explicit error propagation across layers, and the error propagation is implicit due to forward connectivity as shown in Figure 1.

### 2.2 Deriving a biologically plausible rule for the HSIC bottleneck

In this work, we seek to derive biologically plausible rule for optimizing Equation (5). Computing this quantity requires a batch of *N* samples, but we want a rule that operates on single samples arriving sequentially over time. So, we will make a minor notational change to the indexing in Equation (5) for clarity. Our indices will range over {0, −1, …, −(*N*−1)} instead of {1, 2, …, *N*}, so that *x*_0_ refers to the current input sample, *x*_−1_ refers to the previous input sample, etc. We operate on *N* samples, but we are explicit that these samples arrive at different points in time.

Now, we take the gradient of $L_{\text{HSIC}}$ with respect to **θ**^ℓ^ and applying gradient descent. Doing this, we obtain the following update rule:


(6) $$\begin{array}{l} \Delta \theta^{\ell }\propto \nabla_{\theta^{\ell }}{ℒ}_{HSIC}=\frac{1}{{\left(N-1\right)}^{2}}\sum_{p=0}^{-(N-1)}\left[\bar{k}(x_{p},x_{p})-\bar{k}(y_{p},y_{p})\right]\\ \;\nabla_{\theta^{\ell }}\bar{k}(z_{p}^{\ell },z_{p}^{\ell })\\ \;\nabla_{\theta^{\ell }}\bar{k}(z_{p}^{\ell },z_{p}^{\ell })=\frac{2}{N\sigma^{2}}\text{​​}\sum_{n=0}^{-(N-1)}\text{​​}k(z_{p}^{\ell },z_{n}^{\ell })(z_{p}^{\ell }-z_{n}^{\ell })(\nabla_{\theta^{\ell }}z_{p}^{\ell }-\nabla_{\theta^{\ell }}z_{n}^{\ell })\\ \;=\frac{2}{N\sigma^{2}}\text{​​}\sum_{n=1}^{-(N-1)}k(z_{p}^{\ell },z_{n}^{\ell })(z_{p}^{\ell }-z_{n}^{\ell })(\text{​}\nabla_{\theta^{\ell }}f(\theta^{\ell },z_{p}^{\ell -1})\\ \;-\nabla_{\theta^{\ell }}f(\theta^{\ell },z_{n}^{\ell -1})) \end{array}$$

where $\nabla_{\theta^{\ell }}f\left(\theta^{\ell },{\text{z}}_{p}^{\ell -1}\right)$ describes how the post-synaptic activity varies as a function of pre-synaptic activity. We call *N*, the batch size in the deep learning, the *effective batch size* in our work. This rule is similar to the basic rule in Pogodin and Latham (2020), except that they replace $\bar{k}\left({\text{x}}_{p},{\text{x}}_{p}\right)$ with $\bar{k}\left({\text{z}}_{p},{\text{z}}_{p}\right)$ and do not use a centered kernel matrix.

Without modifications, Equation (6) is not biologically plausible. $\nabla_{\theta^{\ell }}\bar{k}\left({\text{z}}_{p}^{\ell },{\text{z}}_{p}^{\ell }\right)$ cannot be called Hebbian when *p* is not equal to zero, since it depends on non-local information from the past. We solve this by making a simplifying approximation. We assume that $\nabla_{\theta^{\ell }}f\left(\theta^{\ell },{\text{z}}_{p}^{\ell -1}\right)=0$ when *p*≠0. In other words, the weights at the current time step do not affect past outputs. With this assumption, we find that


$$\begin{array}{l} \;\nabla_{\theta^{\ell }}\bar{k}(z_{p}^{\ell },z_{p}^{\ell })\\ =\{\begin{array}{ll} \frac{2}{N\sigma^{2}}\sum_{n=1}^{-(N-1)}k(z_{0}^{\ell },z_{n}^{\ell })(z_{0}^{\ell }-z_{n}^{\ell })\nabla_{\theta^{\ell }}f(\theta^{\ell },z_{0}^{\ell -1}) & p=0\\ \frac{2}{N\sigma^{2}}k(z_{0}^{\ell },z_{p}^{\ell })(z_{0}^{\ell }-z_{p}^{\ell })\nabla_{\theta^{\ell }}f(\theta^{\ell },z_{0}^{\ell -1}) & p\neq 0 \end{array} \end{array}$$

Notably, the local term, $\nabla_{\theta^{\ell }}f\left(\theta^{\ell },z_{0}^{\ell -1}\right)$, does not depend on the summation indices and can be factored out. This leads us to our final three-factor update:


(7) $$\begin{array}{l} \Delta W^{\ell }\propto \beta ⊙\xi \\ \;\beta =\nabla_{\theta^{\ell }}f(\theta^{\ell },z_{0}^{\ell -1})\\ \;\xi =\frac{2}{\sigma^{2}N{\left(N-1\right)}^{2}}(\sum_{p=1}^{-(N-1)}[\bar{k}(x_{p},x_{p})-\bar{k}(y_{p},y_{p})]\alpha (z_{p}^{\ell })+\\ \;\sum_{p=1}^{-(N-1)}\alpha (z_{n}^{\ell })[\bar{k}(x_{0},x_{0})-\bar{k}(y_{0},y_{0})])\\ \alpha (z_{p}^{\ell })=k(z_{0}^{\ell },z_{p}^{\ell })(z_{0}^{\ell }-z_{p}^{\ell }) \end{array}$$

Note that β is now a local term that only depends on the current pre- and post-synaptic activity. ξ is a modulating term that adjusts the synaptic update layer-wise. This establishes a three-factor learning rule for Equation (5). In the next section, we discuss how ξ, despite appearing complex, is easy to compute using an auxiliary network of neurons.

To understand the behavior of our rule, we begin by focusing on $\alpha \left({\text{z}}_{p}^{\ell }\right)$. This term drives together the layer representations ${\text{z}}_{0}^{\ell }$ and ${\text{z}}_{p}^{\ell }$, but the strength is weighted by the similarity kernel, $k\left({\text{z}}_{0}^{\ell },{\text{z}}_{p}^{\ell }\right)$. This similarity drive is modulated by the term $\bar{k}\left({\text{x}}_{p},{\text{x}}_{p}\right)-\gamma \bar{k}\left({\text{y}}_{p},{\text{y}}_{p}\right)$. Note that we are specifically focused on the diagonal terms of the centered kernel matrices. This takes a special form:


$$\bar{k}\left({\text{x}}_{p},{\text{x}}_{p}\right)=1-\frac{1}{N}\overset{-\left(N-1\right)}{\underset{n=0}{\sum }}k\left({\text{x}}_{p},{\text{x}}_{n}\right)$$

The summation measures the average similarity of **x**_*p*_ to other samples. As a result, $\bar{k}\left({\text{x}}_{p},{\text{x}}_{p}\right)$ acts as a “surprise” signal. If **x**_*p*_ is identical to all other samples, then $\bar{k}\left({\text{x}}_{p},{\text{x}}_{p}\right)=0$. Conversely, if **x**_*p*_ is unlike any other sample, then $\bar{k}\left({\text{x}}_{p},{\text{x}}_{p}\right)\to 1$. When taken together, the full term $\bar{k}\left({\text{x}}_{p},{\text{x}}_{p}\right)-\gamma \bar{k}\left({\text{y}}_{p},{\text{y}}_{p}\right)$ measures surprise along a decision boundary. If both the input and output is surprising or not surprising, this term goes to zero (where the relative surprise signals are balanced by γ). On the other hand, if the input is surprising, but the output is not, then the similarity drive of $\alpha \left({\text{z}}_{p}^{\ell }\right)$ is strengthened. In effect, this compresses away the differences in the input distribution and more closely matches the desired output. For the opposite case, when the input is not surprising, but the output is, the sign is reversed on $\alpha \left({\text{z}}_{p}^{\ell }\right)$. As expected, this forces the layer to drive the output representations apart.

#### 2.2.1 Computing the modulating signal with an auxiliary network

In order to compute ξ in Equation (7), we require a neural circuit capable of storing information for future use. Recurrent networks can provide such functionality, and Sussillo and Abbott (2009) demonstrate how a reservoir network can be trained to compute complex signals using a local update rule.

For each layer, we construct an auxiliary network of rate-encoded leaky-integrate neurons whose dynamics are governed by Equation (8)


(8) $$\begin{array}{l} \tau_{r}\frac{\text{d}u_{r}}{\text{d}t}=-u_{r}+\lambda W_{r}r+W_{i}r_{i}+W_{\text{fb}}r_{o}\\ \;r=\tanh(u_{r})\\ \;r_{o}=W_{o}u_{r} \end{array}$$

where **u**_*r*_ are the recurrent neuron membrane potentials, **r**_*i*_ is the input signal activity, and **r**_*o*_ is the readout activity. λ is a hyper-parameter that controls the chaos level of the recurrent population.

Following Sussillo and Abbott (2009), we train the auxiliary network using a least mean squares (LMS) FORCE learning rule shown below in Equation (9) (which is a local update),


(9) $$\begin{array}{l} \Delta {\text{W}}_{o}\propto \left({\text{r}}_{o}-\xi \right){\text{r}}^{⊤} \end{array}$$

where ξ is the *true* global error signal in Equation (7).

Training of the reservoir can be done *a priori* so that the reservoir is fixed during the primary learning phase. Alternatively, we can also compute ξ without needing to train the auxiliary network. Instead, a set of buffers can be constructed to remember that last *N* inputs, targets, and current layer's activity. This can be achieved using a delay line circuit where populations of neurons are connected with the appropriately chosen synaptic delay. Given the output of these buffers, computing ξ is trivial since $\bar{k}$, α, and the summation are all implementable functions using neurons.

**Figure 2:**
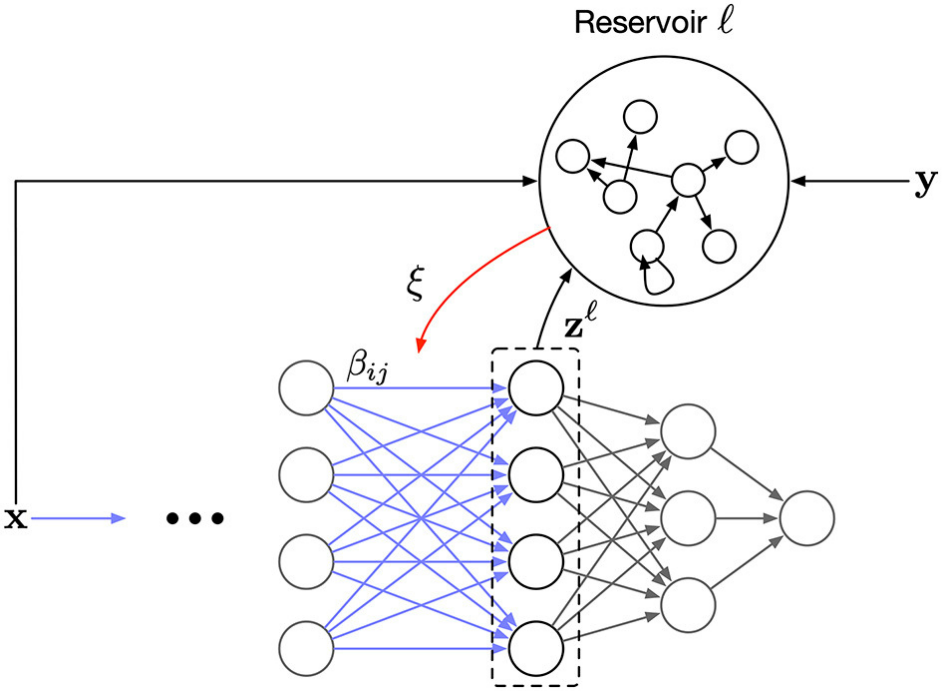
The overall network architecture. Each layer has a corresponding auxiliary reservoir network. The synaptic update, β in Equation (7), is modulated by a layer-wise error signal, ξ, that is the readout from the reservoir.

Figure 2 illustrates the full design of the proposed learning scheme. The reservoir serves as a working memory where the capacity of the memory determines the effective batch size. To the best of our knowledge, our rule is the first to modulate the Hebbian updates of a synapse based on past information stored in a working memory. Furthermore, having a controllable effective batch size means we can study the effect of memory capacity on the learning convergence.

#### 2.2.2 Extensions to convolutional neural networks and spiking neural networks

When feasible, we use fully-connected layers in this work, because they are biologically plausible. Unfortunately, the best performing artificial networks for visual tasks utilize convolution layers. A biologically plausible implementation of convolution itself is an open topic of research (Pogodin et al., 2021). Still, we can support convolutional layers in our rule, since the local term β in Equation (7) is agnostic to the layer function, *f*. The resulting update is not biologically plausible, but any issues stem from *f*, not the learning rule.

Similar to how we extend our rule to convolution neural networks, we can extend it to other network types including spiking neural networks. For spiking neural networks, the primary challenge is the non-linearity of the spike threshold function. Standard techniques such as surrogate gradients or probabilistic threshold functions can be used to correctly derive β (Neftci et al., 2019). Another important feature of SNNs is recurrence or feedback connections. Traditionally, this is handled by back-propagation-like algorithms by unrolling the network evaluations over time and treating the temporal dimension spatially. A similar approach could be done with our rule, but unlike back-propagation through time, the HSIC update does not introduce coupling across timesteps. In this way, we are able to remain biologically plausible even in the presence of recurrence. Yet, when dealing with recurrent connections, the stability of weight updates is always a concern. It is not immediately clear that our rule as given should be stable. The analysis and potential augmentations to our approach for recurrent networks is out of the scope of this paper, and we leave it for future work.

## 3 Results

We test our approach on a variety of synthetic and benchmark datasets. The code to reproduce each experiment is available at https://github.com/darsnack/biological-hsic/ along with instructions. Since our method processes samples one at a time, training networks can be a computationally intensive process. To make experimentation tractable, our evaluation is broken down into three stages:

1. We test the ability for a reservoir network to learn to compute the global modulatory signal, ξ, in Equation (7) as describe in Section 2.2.1.
2. We train multi-layer networks of rate-encoded leaky-integrate neurons on small synthetic datasets. We avoid simulating the reservoir for computational efficiency.
3. We train deep multi-layer networks of artificial neurons on larger scale machine learning benchmark datasets. We use artificial neurons instead of biological neurons for computational efficiency.

### 3.1 Reservoir experiments

First, we verify the ability for the reservoir to reproduce the true signal ξ in Equation (7). We use a recurrent population of 2,000 leaky-integrate neurons with τ_*r*_= 5 ms and λ = 1.2 (as described in Section 2.2.1). For the input and output signals, we use a hundred random samples from MNIST. Corresponding random hidden activation signals, *Z*∈ℝ^10 × 100^, are drawn from Unif(0, 1). Each sample is presented to the network for 10 ms and the network is trained for 10 epochs. For evaluation, we generate a hundred new inputs and process them with the network with all parameters fixed (i.e., no learning). A complete list of experimental parameters is in Table 2.

**Table 2:** Reservoir experiment parameters.

**Parameter name**	**Symbol**	**Value**
Simulation time step	Δ*t*	1 ms
Neuron time constant	τ_*r*_	5 ms
Sample time constant	Δ*t*_sample_	20 ms
Reservoir recurrent strength	λ	1.2
Effective batch size	*N*	10
HSIC balance parameter	γ	2
HSIC scale parameter	σ	0.5
Learning rate	η	5 × 10^−4^
Num. of epochs	*T*	10

**Figure 3:**
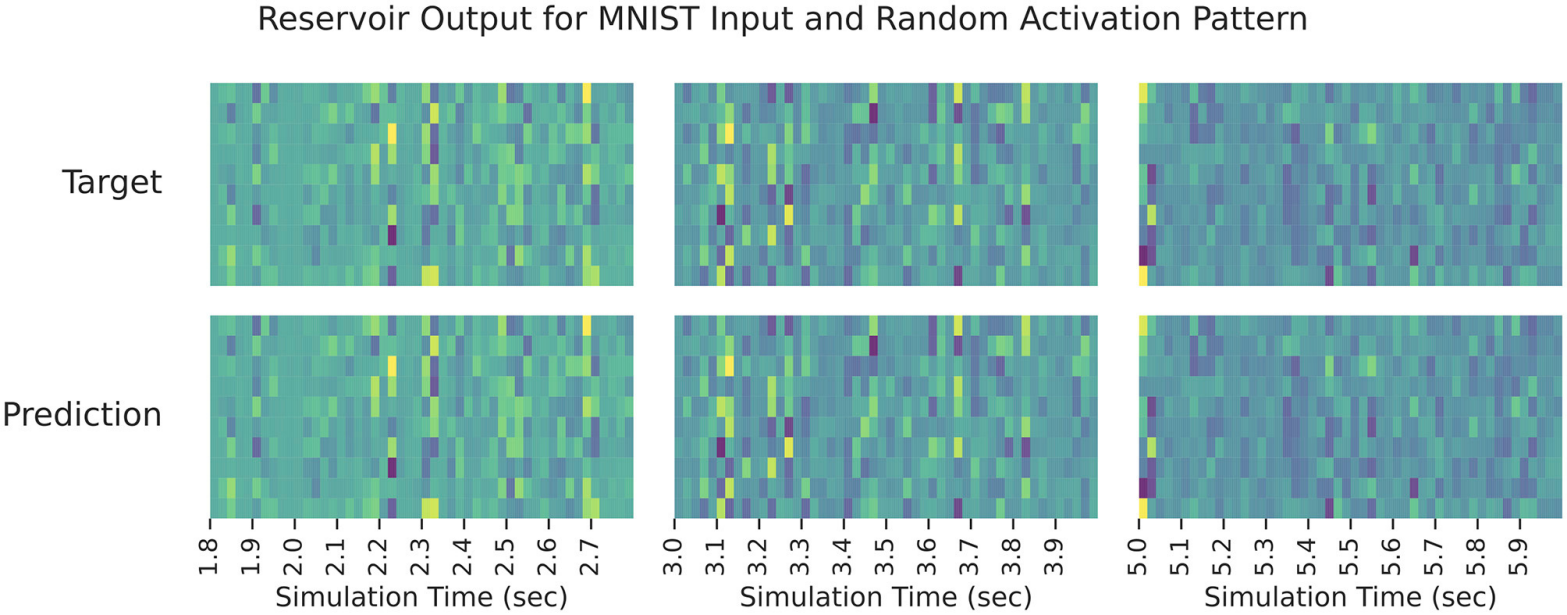
The reservoir output when learning ξ in Equation (7). Each sub-panel column displays the output during a different time interval of testing. The rows in a given sub-panel correspond to each element of true/predicted ξ. In all cases, the predicted output signal matches the target signal closely.

The results can be seen in Figure 3 which shows the reservoir output for all elements of the predicted modulatory signal against the target modulatory signal, ξ. The network is able to close match the target shortly after learning begins, and it is able to persist its performance long after learning stops. Note that this demonstrates how the auxiliary network can be pre-trained to compute ξ—it is not learning a data-specific computation, it is learning how to buffer *N* samples of its input and post-process them to compute ξ.

### 3.2 Small dataset experiments

Now, we show that our learning rule is capable of training multi-layer networks of leaky-integrate neurons to solve “small scale” tasks. For computational efficiency, we no longer simulate the reservoir network, but compute its readout, ξ, directly. All networks in this set of experiments use the following neuron model in Equation (10):


(10) $$\begin{array}{l} \tau_{m}\frac{\text{d}u^{\ell }}{\text{d}t}=-u^{\ell }+W^{\ell }z^{\ell -1}+b^{\ell }\\ \;z^{\ell }=\text{relu}(u^{\ell }) \end{array}$$

where τ_*m*_ is the neuron time constant, and **W**^ℓ^ and **b**^ℓ^ are parameters. A full description of the experimental parameters is in Table 3.

**Table 3:** Small dataset experiment parameters.

**Parameter name**	**Symbol**	**Value**
Simulation time step	Δ*t*	1 ms
Neuron time constant	τ_*m*_	5 ms
Sample time constant	Δ*t*_sample_	20 ms
Effective batch size	*N*	64
HSIC balance parameter	γ	10
HSIC input scale parameter	σ_*x*_	0.3
HSIC layer scale parameter	σ_*z*_	2
HSIC output scale parameter	σ_*y*_	0.25
Learning rate	η	1 × 10^−4^
Num. of epochs	N/A	25
Num. of random seeds	N/A	30

**Figure 4:**
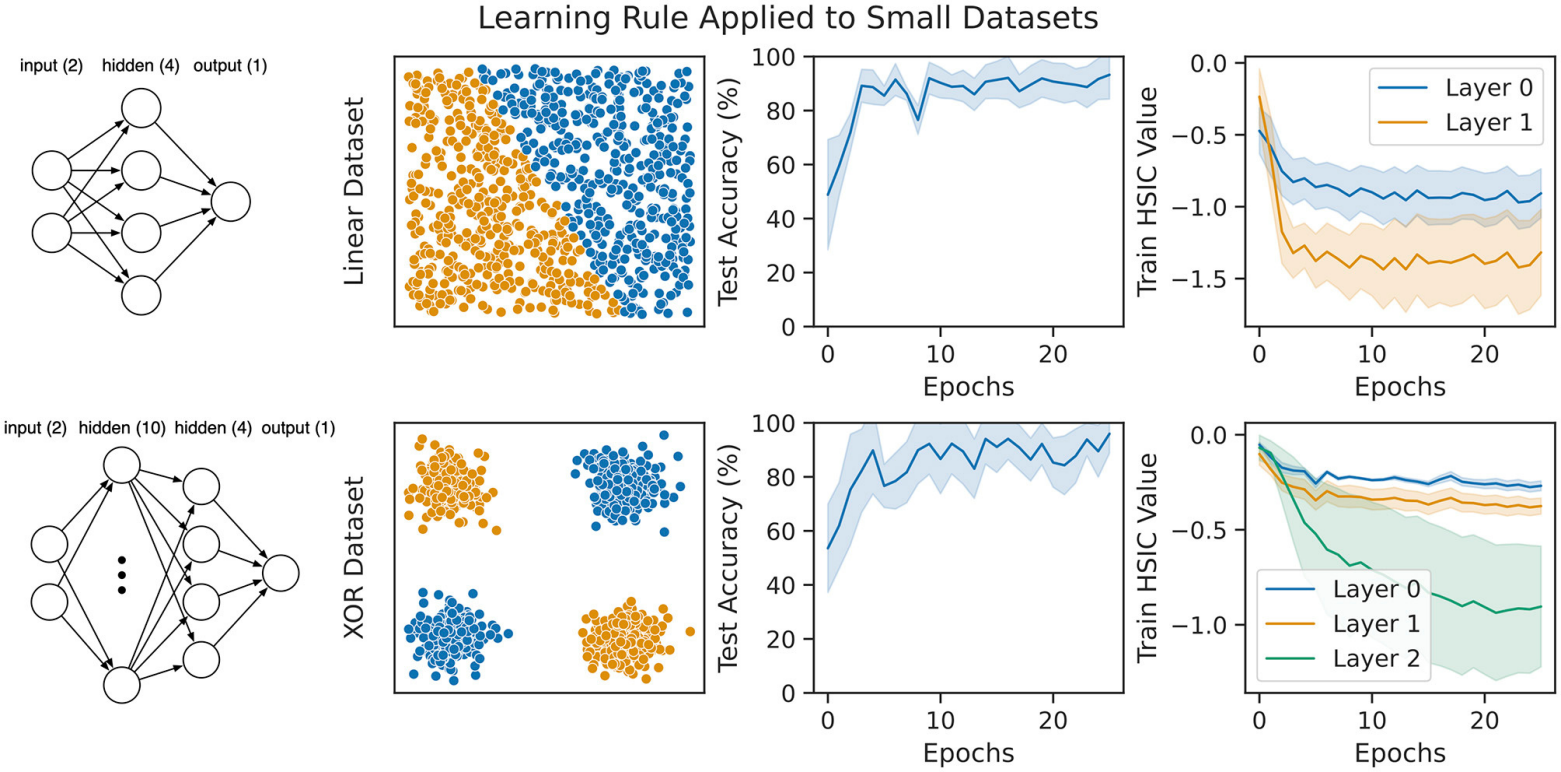
Our learning rule applied to two synthetic datasets with multi-layer feedforward networks of leaky-integrate neurons (see Equation 10). **(Top)** A linearly separable dataset drawn from Unif([0, 1] × [0, 1]). The network trained on this dataset consists of a single hidden layer with four neurons. **(Bottom)** A non-linear XOR dataset with small Gaussian noise added around each input cluster. The network trained on this dataset consists of two hidden layers of size ten and four, respectively. For both datasets, our models converge to nearly 100% accuracy, and this coincides with decreasing the HSIC bottleneck at every layer.

We consider two synthetic datasets consisting of a thousand samples each (a hundred samples are held out for testing). First, we generate a linearly separable set of labeled points drawn from Unif([0, 1] × [0, 1]) and train a network with a single hidden layer on them [shown in Figure 4 (top left)]. Since only a linear classifier is required to predict this dataset, we also generate a non-linear XOR dataset and train a network with two hidden layers on it [shown in Figure 4 (bottom left)]. The final layer of both networks is trained against a task-specific cross-entropy objective. The results on both datasets are shown in Figure 4. Within a few epochs, our rule is able to achieve nearly 100% accuracy on both datasets. Additionally, we also show the value of the HSIC bottleneck (Equation 5) in the rightmost column of Figure 4 during training. This demonstrates that our rule does reduce the HSIC bottleneck across all layers, and this reduction corresponds to an improvement in test accuracy on the task.

### 3.3 Large dataset experiments

Finally, we test our rule on two standard machine learning benchmark vision datasets—MNIST and CIFAR-10. We use feedforward networks of artificial neurons with a ReLU activation function (this is done to keep our experiments tractable). Our rule is compared against a back-propagation baseline.

We use a multi-layer fully-connected network on MNIST with architecture FC(128) → FC(64) → FC(10). For CIFAR-10, we use a convolutional neural network with architecture Conv(3 × 3 × 128) → Avg. Pool(2) → Conv(3 × 3 × 256) → Avg. Pool(2) → FC(10). Both the baseline and our rule are trained using an Adam optimizer with default hyper-parameters. A complete list of experiment parameters is in Table 4.

**Table 4:** Large scale experiment parameters.

**Parameter name**	**Symbol**	**MNIST value**	**CIFAR-10 value**
Back-propagation learning rate	N/A	1 × 10^−3^	5 × 10^−4^
Our rule learning rate	N/A	1 × 10^−4^	5 × 10^−5^
Effective batch size	*N*	8	4
HSIC balance parameter	γ	2	50
HSIC input scale parameter	σ_*x*_	0.5	1
HSIC layer scale parameter	σ_*z*_	1	0.5
HSIC output scale parameter	σ_*y*_	0.1	0.1
Num. of epochs	N/A	25	50
Num. of random seeds	N/A	30	15

**Figure 5:**
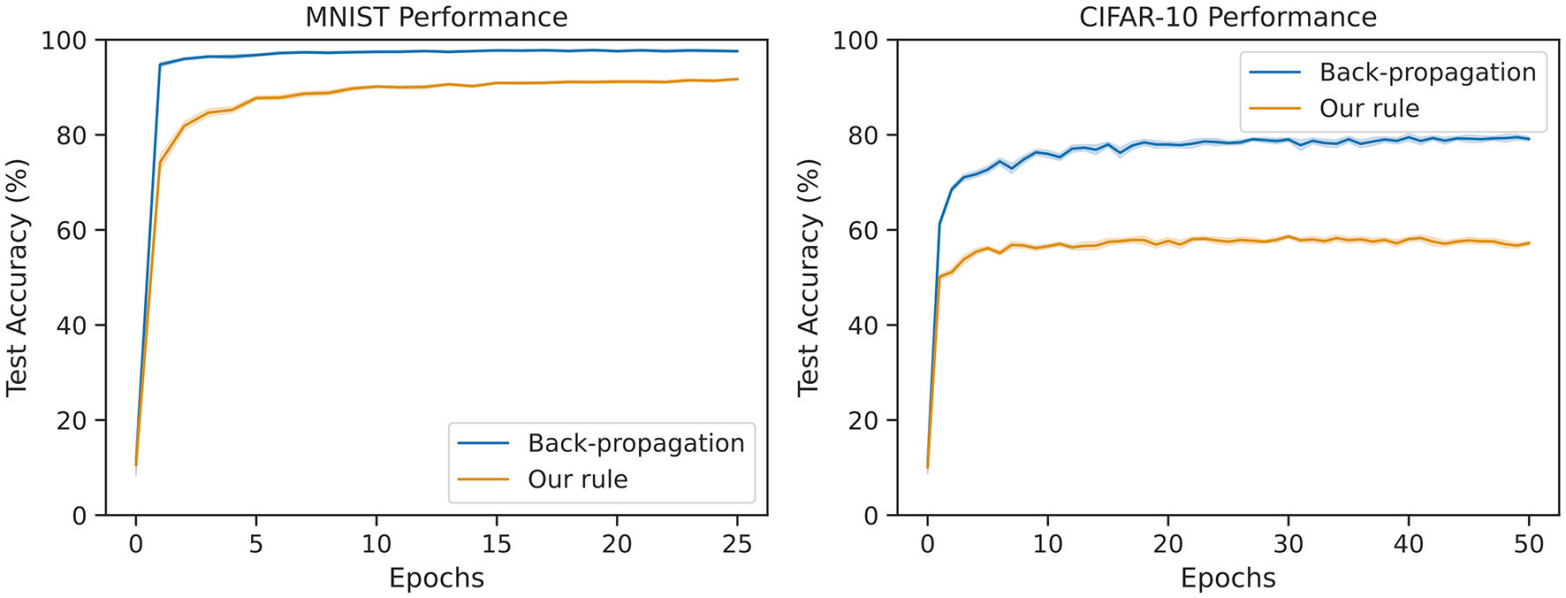
Average test accuracy over many trials on MNIST and CIFAR-10 for back-propagation and our method. The MNIST network is an MLP with 128 and 64 hidden neurons. The CIFAR-10 network is a CNN with 128 and 256 features followed by a single fully-connected output layer. Our rule reaches above 91% accuracy on MNIST (within 7% of the baseline). Our rule reaches around 61% accuracy on CIFAR-10 (within 19% of the baseline).

Figure 5 shows the test performance over the course of training. Even though learning occurs more slowly, our rule reaches comparable the performance with the back-propagation baseline. While the gap between back-propagation and our rule widens on CIFAR-10, it does reach 60% test accuracy which is higher than training just the last layer (this reaches only 39%). Given that each layer in our method has no explicit information about the performance of other layers, the fact that hierarchical learning is possible is remarkable.

### 3.4 Effects of memory capacity

**Figure 6:**
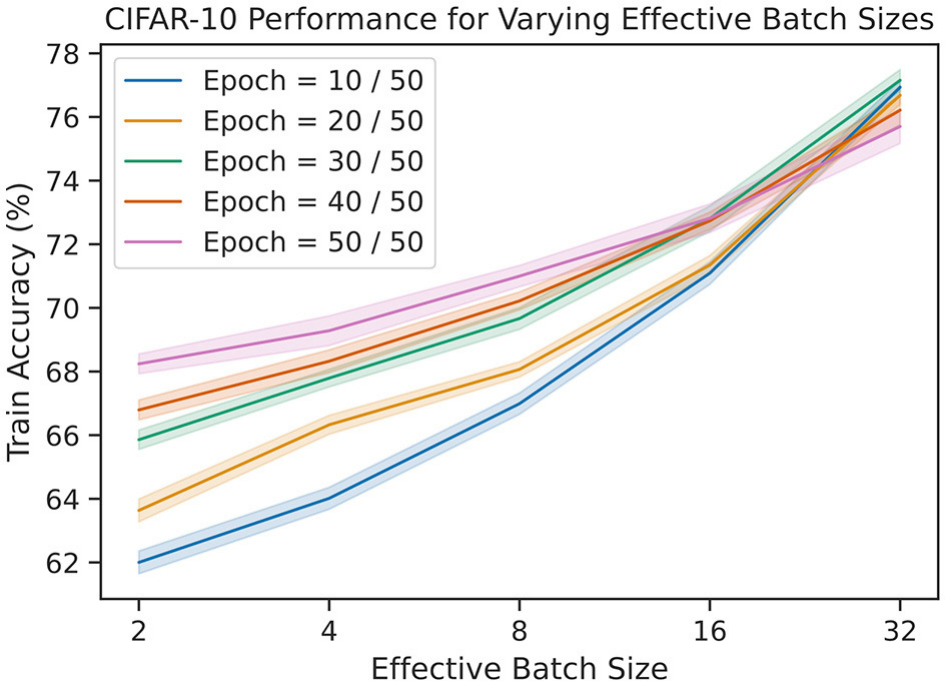
The train accuracies on CIFAR-10 for varying number of epochs and effective batch sizes. We see that accuracy improves logarithmically as a function of batch size.

One of the novel features of our rule is the ability to control the memory capacity of the update. To explore this parameter, we repeat the same CIFAR-10 experiments as before for various effective batch sizes. The results are shown in Figure 6. Not only does the final training performance increase as a function of batch size, the rate of learning also increases. Unfortunately, this improvement is logarithmic, leading to diminishing returns.

## 4 Discussion

In this work, we proposed a three-factor learning rule for training feedforward networks based on the information bottleneck principle. The rule is biologically plausible, and we are able to scale up to reasonable performance on MNIST. We do this by factoring our weight update into a local component and global component. The local component depends only on the current synaptic activity, so it can be implemented via Hebbian learning. In contrast to prior work, our global component uses information across many samples seen over time. We show that this content can be stored in an auxiliary reservoir network, and the readout of the reservoir can be used to modulate the local weight updates. To the best of our knowledge, this is the first biological learning rule to tightly couple the synaptic updates with a working memory capacity. We verified the efficacy of our rule on synthetic datasets, MNIST, and CIFAR-10, and we explored the effect of the size of the working memory capacity on the learning performance.

Even though our rule does perform reasonably well, there is room for improvement. The rule performs best when it is able to distinguish between different high dimensional inputs. The resolution at which it separates inputs is controlled by the parameter, σ, in the kernel function (Equation 4). The use of a fixed σ is partly responsible for the slow down in convergence in Figure 5. In Ma et al. (2019), the authors propose using multiple networks trained with the different values of σ and averaging the output across networks. This allows the overall network to separate the data at different resolutions. Future work can consider a population of networks with varying σ to achieve the same effect. Addressing the resolution issue will be important for improving the speed and scalability of the learning method.

Additionally, our rule is strongly supervised. While the mechanism for synaptic updates is biologically plausible, the overall learning paradigm is not. Note that the purpose of the label information in the global signal is to indicate whether the output for the current sample should be the same or different from previous samples. In other words, it might be possible to replace the term $\bar{k}\left({\text{y}}_{0},{\text{y}}_{p}\right)$ in Equation (7) with a binary teaching signal. This would allow the rule to operate under weak supervision. Alternatively, we could use contrastive learning, where output distribution, *Y*, is replaced by the output of a different network. Ideally, this other network should process a different, but related modality (e.g., a visual network and auditory network that are trained against each other using a contrastive approach).

Most importantly, while our rule is certainly biologically plausible, it remains to be seen if it is an accurate model for circuitry in the brain. Since rules based on the information bottleneck are relatively new, the corresponding experimental evidence must still be obtained. Yet, we note that our auxiliary reservoir serves a similar role to the “blackboard” circuit proposed in Mumford (1991). This circuit, present in the thalamus, receives projected connections from the visual cortex, similar to how each layer projects its output onto the reservoir. Furthermore, Mumford suggests that this circuit acts as a temporal buffer and sends signals that capture information over longer timescales back to the cortex like our reservoir.

So, while it is uncertain whether our exact rule and memory circuit are present in biology, we suggest that an in-depth exploration of memory-modulated learning rules is necessary. Even in the absence of a biological counter-part, our rule captures important properties necessary for neuromorphic hardware—locality, asynchrony, and real-time processing. We achieve this by suggesting a fundamentally different objective training deep neural networks, in line with recent work. We hope this work prompts further exploration of novel, non-back-propagation-based approaches for learning.

## Data availability statement

Publicly available datasets were analyzed in this study. This data can be found here: http://yann.lecun.com/exdb/mnist/.

## Author contributions

KD derived the theory and learning rule and performed the simulations. ML helped design the experiments and provided feedback on the theory. KD and ML contributed to writing the manuscript. All authors contributed to the article and approved the submitted version.
